# Flooding: another abiotic stressor to consider in plant-insect interactions

**DOI:** 10.3389/fpls.2026.1813020

**Published:** 2026-04-28

**Authors:** Satinderpal Kaur, Esther Ngumbi

**Affiliations:** Department of Entomology, University of Illinois Urbana-Champaign, Urbana, IL, United States

**Keywords:** flooding, insect herbivory, plant secondary metabolites, plant volatile compounds, reoxygenation, plant- insect interactions

## Abstract

Flooding is an increasingly important plant abiotic stress that is intensifying with climate change, yet its ecological implications, particularly for plant- insect interactions remain poorly understood and synthesized. Both flooding and after-flood recovery period disrupt plant growth, physiology, metabolism and biochemistry. Although plant responses to flooding have been extensively studied, the mechanistic links connecting these flooding and after-flood phase induced changes to insect herbivore performance and behavior remain largely unresolved. In this review, we synthesize current knowledge on the pathways through which flooding could potentially re-shape plant-insect interactions. We highlight several interconnected mechanisms, including flooding-induced changes in plant physiology and nutrient status, shifts in phytohormone signaling and plant changes during after-flood recovery phase. These flooding-induced changes can affect insect herbivore growth, performance, oviposition, interactions with natural enemies and ultimately pest outbreaks. Our synthesis reveals that although some individual mechanisms have been documented, the links connecting flooding-induced plant responses to insect outcomes remain largely unresolved. While more foundational evidence regarding flooding-plant-insect interactions is needed, available studies suggest that flooding should be considered as a multi-pathway stress that can reorganize plant metabolic, chemical and nutritional landscape triggering cascading ecological consequences across diverse herbivore guilds. Addressing these gaps will require integrative approaches and will provide a foundation for developing new testable plant- insect interaction hypotheses and advancing ecological theories under changing environmental conditions.

## 1 Introduction

Plants and insect herbivores have co-evolved over millions of years, leading to complex relationships with significant implications for agroecosystem health (Pincebourde et al., 2017; Dofuor et al., 2024). As two of the most diverse and abundant groups of organisms on Earth, plants and insects engage in interactions that directly influence pest pressure, crop yields, and ultimately food security (Fauvel, 1999; Jankielsohn, 2018). Studying these interactions helps us understand how they are shaped not only by evolutionary forces but also by external biotic and abiotic factors like nutrient availability, drought, and flooding. These environmental stresses can disrupt plant–insect relationships, destabilize agroecosystems, and amplify pest risks (Mattson and Haack, 1987; Huberty and Denno, 2004; Tylianakis et al., 2008; DeLucia et al., 2012; Hamann et al., 2021; Kansman et al., 2022). In the face of climate change, where extreme events are expected to grow more intense, it is essential to understand how environmental stress affects plant–insect interactions to develop resilient cropping systems that minimize pest damage, reduce chemical use, and protect food production (Altieri and Nicholls, 1999; Giron et al., 2018; Dofuor et al., 2024).

Research on abiotic stress-plant-insect interactions has resulted in the generation of several influential ecological hypotheses, such as the plant stress hypothesis (White, 1969) and plant vigor hypothesis (Price, 1991) that have shaped our understanding of how environmental stressors impact plant-insect interactions (Dorschner et al., 1986; Tariq et al., 2012; Grinnan et al., 2013; Bauerfeind and Fischer, 2013). Collectively, these studies have highlighted how shifts in plant physiology, metabolism, and chemistry can cascade to impact plant-insect interactions (English-Loeb et al., 1997; Weldegergis et al., 2015; Gao et al., 2020). However, the empirical foundation of these frameworks has relied overwhelmingly on drought as the primary stressor (Rouault et al., 2006; Grinnan et al., 2013; Gely et al., 2020). This drought focus constrains the generality of these theories and has left other emerging stressors unnoticed, most notably flooding. Because flooding is intensifying, its absence from abiotic stress-plant-insect interactions represents a critical knowledge gap in the field of ecology, and it limits our ability to predict plant-insect interaction dynamics in agroecosystems both today and into the future.

In this review, we argue that flooding also deserves attention, and the ways this emerging stressor may affect plant-insect interactions have not been fully explored. Flooding broadly refers to excess water that displaces oxygen in the soil, creating hypoxic conditions that can involve waterlogging, in which part of the plant is submerged or complete submergence, in which the entire plant is underwater (Sasidharan et al., 2017; Fukao et al., 2019). In this review, we use “flooding” as a general term encompassing both waterlogging and submergence, while retaining the original terminology of individual studies when describing specific experimental conditions. Flooding induces a unique set of physiological responses in plants, primarily driven by oxygen limitation in the root zone. Oxygen deprivation limits cellular respiration, reduces photosynthesis, and produces reactive oxygen species such as superoxide and hydrogen peroxide (Voesenek and Sasidharan, 2013; Mustroph, 2018; **Supplementary Table 1**). Additionally, flooding stress in plants occurs in a sequential manner. When floodwaters recede, plants struggle to resume normal growth, leaving them vulnerable to other environmental stresses including insect herbivory (Yeung et al., 2019). It is well established that flooding harms plant growth and can ultimately cause plant death, threatening agricultural crop production and food security worldwide (Ngumbi, 2025; **Supplementary Table 1**). These flooding induced changes can influence plant traits that are critical for herbivore performance, including nutrient content, volatile emissions, and defensive metabolites.

**Figure 1:**
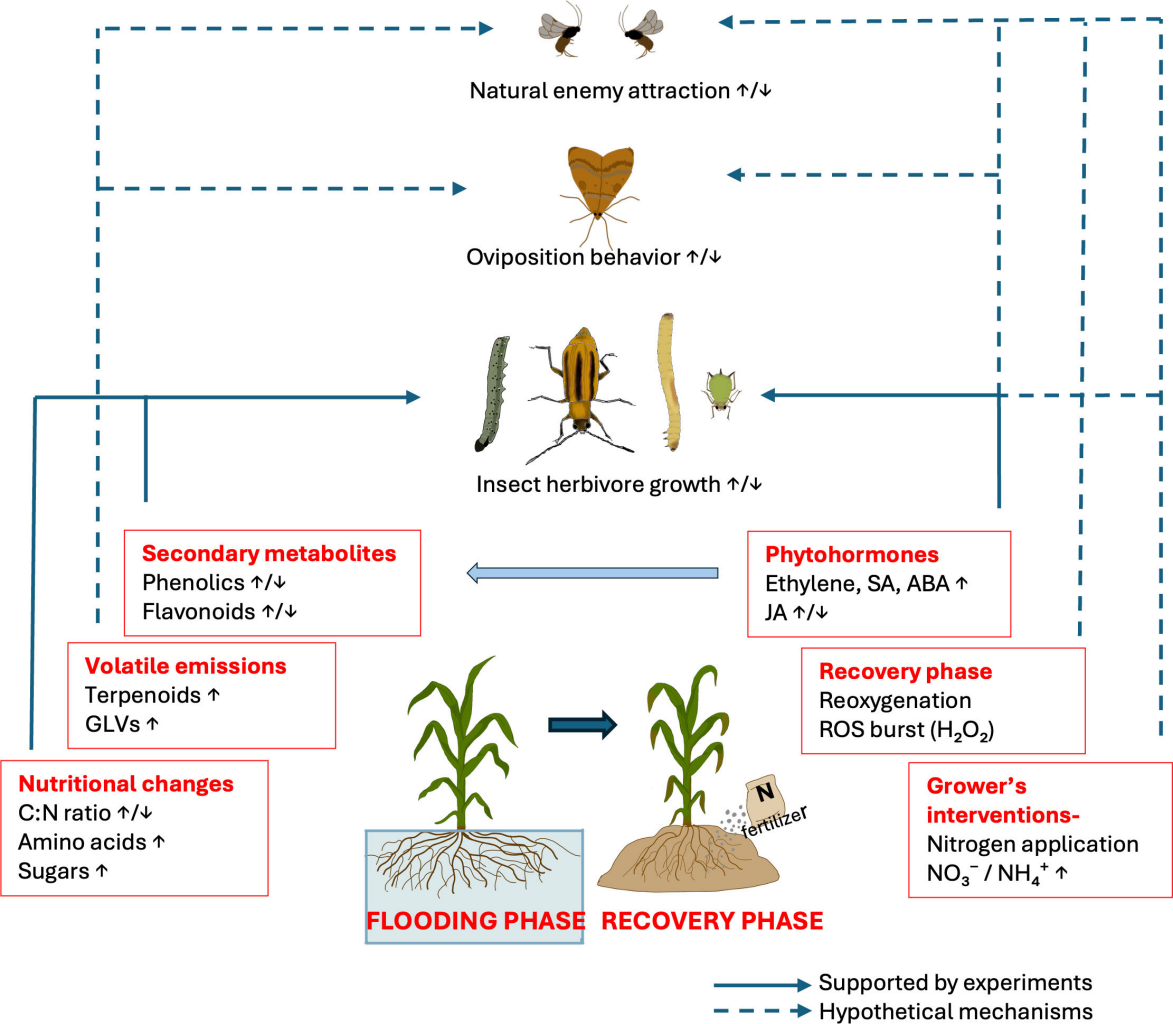
Schematic illustration of the pathways through which flooding and after- flood phase can influence plant-insect interactions.

Although recent advances in flooding research have clarified many important aspects of flood-induced plant changes (**Supplementary Table 1**), we still lack essential information about how these changes influence plant-insect interactions. In this review, we use the term “pathways” to refer to mechanistic routes through which flooding-induced changes in plant physiology may influence plant–insect interactions. These pathways include changes in phytohormonal signaling, plant volatile and non- volatile secondary metabolites production, plant nutritional quality, and through plant changes induced during the after-flood recovery phase. While these pathways are presented separately for clarity, they are not independent processes and may interact with one another (**Figure 1**, **Table 1**). Based on the knowledge gained so far, we emphasize our perspectives on important patterns of flooding-induced plant changes and their relationship with piercing/sucking and chewing insect herbivores, while highlighting the significant gaps that still exist.

**Table 1:** Synthesis of reported effects of flooding stress on insect herbivore performance and associated plant physiological responses.

Sr. No.	Plant species/and insect species	Plant growth stage/Flooding duration	Main objective	Methodology used	Effect of flooding on plant traits	Effect of flooding on insect growth
1 (Xiong et al., 2026)	*Arabidopsis thaliana/Bemisia tabaci and Myzus persicae*	21- day old plants/7 days	To understand why how flooding impairs plant resistance to piercing-sucking insects	Gene expression, phytohormone quantification, Electrical penetration Graph (EPG)	-Ethylene accumulation increased -JA and SA not found to be involved in flooding induced reduction in plant resistance	-Herbivores performed better on flooded plants
2 (Lewis et al., 2025)	Soyabean (*Glycine max)*/soybean aphid (*Aphis glycines*)	14 days old plants/2 days	To study how flooding influences the interactions between soybean and virulence and avirulence biotypes of soybean aphid	Aphid gene expression	NA	-Flooding negatively impact avirulent aphid biotype -the differential response of virulent and avirulent types might be due to differences in gene expression between two biotypes
3 (Gorman et al., 2025)	Maize (*Zea mays*)/*Spodoptera frugiperda*	3-week-old plants/3 days	To verify that flavonoids are involved in maize response to combined flooding and herbivory	RNAseq and metabolic profiling	-Increase in flavonoids and SA accumulation	-the flavonoid deficient mutant was found compromised in flooding- induced resistance against *Spodoptera frugiperda*, and also in Salicylic acid induction indicating SA and flavonoids induced defense against *S. frugiperda* under flooding conditions.
4. Mleziva and Ngumbi, 2024	Maize (*Zea mays*) and Teosinte (*Zea nicaraguensis*)/ *Spodoptera frugiperda*	14-day old plants/7 days	To study the influence of flooding and herbivory stress on defensive secondary metabolite production	Metabolomic profiling and headspace volatile collection	- Teosinte and maize showed variation in the flooding induced volatiles and secondary metabolites -Differential accumulation of Flavonoids in plant roots during flooding and herbivory for maize and teosinte	NA
5.Kamps and Poelman, 2024	3 species of *Rorippa* genus/ Aphids*- Myzus persicae*, *Lipaphis erysimi*, and caterpillars: *Pieris brassicae*, *Plutella xylostella*	NA	To understand the influence of waterlogged and drought conditions on the plant resistance against insects	NA	NA	-Aphids performed worse on waterlogged conditions than drought plants -the caterpillars were not affected by water availability for their host plant.
6. Ngumbi et al., 2022	Tomato (*Solanum lycopersicum*)/ *Spodoptera exigua*	5-week-old plants/NA	To study influence of flooding, herbivory and their combination on the volatile emission composition and gene expression	Solid phase micro-extraction volatile collection and gene expression	-Flooding downregulated genes associated with cytokinin catabolism and general defense response and upregulated genes associated with ethylene biosynthesis, anthocyanin biosynthesis, and gibberellin biosynthesis	NA
7. Ngumbi and Ugarte, 2021	Maize (*Zea mays*)/g *Spodoptera frugiperda*	18-day old plants/7 days	To study the influence of flooding, herbivory and their combination on the volatile emission composition	Solid phase micro-extraction volatile collection	-combination of flooding and herbivory resulted in higher emission of volatiles than individual stresses	NA
8 (Lee et al., 2020)	*Arabidopsis thaliana*/*Pieris rapae*	10-days old seedlings/1–48 hours	To study the impact of submergence on plant defense system against insect herbivores	Transcriptomics	-flooding deactivates wound-induced defense against herbivore attack and reduced expression of jasmonic acid (JA) biosynthesis	-flooding increased larval growth
9. Block et al., 2020	Maize (*Zea mays*)/*Spodoptera frugiperda*	2-week-old plants/3 days	To study the plant response to the combined stress of flooding and herbivory	Metabolomics, gene expression	- Combined stress led to elevated production of defensive hormones- Salicylic acid	- flooding reduced larval growth
10. Nguyen et al., 2016	*Solanum dulcamara/Spodoptera exigua*	30 days old plants/5 days	To compare the effects flooding and drought on plant resistance against insect herbivores	Transcriptomics and metabolomics	-flooding increased Abscisic acid and Salicylic acid	-flooding increased larval growth
11 (Tindall et al., 2013)	Rice (*Oryza sativa*)/rice water weevil (*Lissorhoptrus oryzophilus)*	31 to 51 days old plants based on different locations/deep and shallow flooding treatments	to determine if the depth of flooding would impact numbers of *L. oryzophilus* on rice plants under field conditions	Insect sampling	NA	-During all sampling dates across all locations, fewer insect larvae were collected from plants in shallow-flooded plots as compared to deep- flooded plots.
12 (Stout et al., 2002)	Rice (*Oryza sativa*)/rice water weevil (*Lissorhoptrus oryzophilus)*	4–17 days old plants/deep and shallow flooding treatments	To test the influence of weevil oviposition preference to different depths of flooding of plants	Oviposition assays	NA	- female weevils showed ovipositional preference for plants deep flooded to a depth of 10.2 cm over unflooded plants and shallow flooded plants -adults weevils preferred flooded plants for feeding

## 2 Pathways through which flooding can affect plant-insect interactions

### 2.1 Pathway 1: Flooding-induced changes in phytohormones

Plants have evolved a sophisticated phytohormone-based system of signaling to ensure early detection of insect herbivore attack and induce efficient defenses to inhibit them before any extensive loss (Howe et al., 1996; Hettenhausen et al., 2015). Interestingly, beyond inducing plant defense responses against insects, phytohormones such as JA, SA, ABA, and ethylene are essential for fine-tuning plant physiological processes that help plants grow under stressful environmental conditions (Bailey-Serres et al., 2012; EL Sabagh et al., 2022).

#### 2.1.1 Mechanisms of flooding-induced changes

Flooding profoundly alters phytohormone production and signaling in plants through multiple mechanisms. Due to limited gas exchange, plants trap ethylene within tissues, elevating its accumulation. As a result, ethylene acts as a primary flooding signal, driving adaptive responses such as adventitious root formation, internode elongation, and aerenchyma development by activating ethylene response factors (ERFs) (Bailey-Serres et al., 2012; Khan et al., 2020). The elevation of ethylene is known to inhibit ABA biosynthesis by suppressing ABA-related gene expression, and, in turn, reduced ABA levels are found to enhance plant adaptation to flooding stress by facilitating shoot elongation (De Ollas et al., 2021; Han et al., 2025). Apart from ABA and ethylene, JA and SA are also positive players in plant response to flooding by quenching flooding-induced ROS and by regulating root architecture (Wang and Komatsu, 2022).

#### 2.1.2 Emerging patterns associated with this pathway

Flooding was found to increase the accumulation of ABA and SA but failed to increase the accumulation of JA in *Solanum dulcamara*, resulting in increased growth of the chewing herbivore *Spodoptera exigua* (Nguyen et al., 2016). In contrast, flooding reduced the growth of the herbivore *Spodoptera frugiperda* in maize (*Zea mays)*. It was found that increased insect resistance in flooded maize might result from a dramatic increase in SA in plants experiencing both stresses (flooding and herbivory) (Block et al., 2020). To further support this hypothesis, the researchers utilized a loss-of-function mutant in the maize homologue of SA receptor NPR1. On comparing the insect growth on flooded and non-flooded plants of this mutant (npr1), no significant differences in insect growth were found. Hence, the flood-induced accumulation of SA in maize plants increased resistance to insect herbivores (Block et al., 2020). Moreover, the effect of flooding on piercing and sucking insect herbivores (*Bemisia tabaci* and *Myzus persicae*) was tested in *Arabidopsis thaliana*, where flooding increased ABA accumulation and increased the performance of insect herbivores, with no influence of JA or SA accumulation (Xiong et al., 2026). On the contrary, another study testing *Arabidopsis thaliana found that* flooding with physical water flow upregulated key transcripts involved in SA and JA synthesis. Interestingly, this effect was more pronounced for flooding with physical flow (Kaji et al., 2024). From these studies, it is evident that flooding yields mixed results, showing varied accumulation of phytohormones, differentially influencing insect herbivores by species and feeding guild, and thus warrants further investigation.

These studies offer critical foundational evidence for the potential effects of flooding on phytohormone signaling in plants, which can have ecological consequences on larger scale. Considering the enormous role of plant hormones in mediating a wide range of plant interactions, it is crucial to understand how flooding can alter their accumulation in key crop species and how this accumulation is linked to plant responses to insect herbivory.

### 2.2 Pathway 2: Flooding-induced changes in volatile and non-volatile plant secondary metabolites

#### 2.2.1 Non- volatile plant secondary metabolites

Plants have developed a wide variety of specialized “secondary” metabolites to protect themselves against environmental stresses, including insect herbivory (Fraenkel, 1969; Bennett and Wallsgrove, 1994; Singh et al., 2021; Zhao et al., 2025). Importantly, beyond defending against insect herbivory, these metabolites are also activated during abiotic stresses, including flooding, to help mitigate stress (Akula and Ravishankar, 2011; Kumar et al., 2023).

##### 2.2.1.1 Mechanisms of flooding- induced changes

Various mechanisms can influence how flooding affects the production of secondary metabolites. During flooding, oxygen deprivation impacts cellular, molecular, biochemical, physiological, morphological, and metabolic processes (Tamang and Fukao, 2015; Zhou et al., 2020). Along with these processes, oxygen deprivation dramatically shifts the soil microbial community from aerobic, plant growth-promoting rhizobacteria (PGPR) to anaerobic microbial communities. Such changes could potentially influence PGPR-mediated priming of plant defenses, such as metabolic reprogramming characterized by dynamic changes in flavonoids and glycoalkaloids, among others (Mhlongo et al., 2020). Additionally, limited oxygen for oxidative phosphorylation during respiration forces plants to use anaerobic pathways to produce ATP, thereby changing carbon and energy allocation and altering nutrient composition and redirecting metabolite biosynthetic pathways (Coutinho et al., 2018). Furthermore, hypoxic conditions lead to the accumulation of reactive oxygen species (ROS), such as superoxide (O_2_^•−^) and hydrogen peroxide (H_2_O_2_), which can damage plant cells (Manik et al., 2019; Xiao et al., 2020). To neutralize these ROS and protect cells, plants activate the phenylpropanoid biosynthetic pathway to produce phenolic compounds such as flavonoids, which are strong antioxidants and help alleviate stress (Kumar et al., 2020; Umićević et al., 2024). Another key mechanism involves changes in hormonal regulation during flooding. For instance, the accumulation of ethylene, jasmonic acid, and salicylic acid can directly regulate and modulate inducible defensive metabolites such as phenolics and alkaloids (Sasidharan et al., 2018; Pérez-Llorca et al., 2023).

##### 2.2.1.2 Emerging patterns associated with this pathway

A few studies have begun exploring the impact of flooding on plant secondary metabolite production. Mleziva and Ngumbi (2024) examined above-ground and below-ground metabolic changes in maize (*Zea mays*) and its wild relative, teosinte (*Zea nicaraguensis*), under flooding and herbivory (*Spodoptera frugiperda*) stress. They observed that flooding, both individually and in combination with herbivory, increased phenolic concentrations in both species. They also tested for below-ground metabolic changes and reported significant differences in the flavone and flavonol biosynthetic pathways between flooded and control teosinte roots, with the flavanone naringenin and the flavones apigenin and luteolin accumulating in the roots due to flooding. Similarly, flooding (waterlogging) induced accumulation of flavonoids were found in soybean (*Glycine max*) leaves (Adegoye et al., 2023); *Chrysanthemum morifolium* (Wang et al., 2019*)* and Quinoa (Li et al., 2026). However, contrary to these studies, in soybean roots, flooding was found to reduce the accumulation of isoflavones as compared to control (Coutinho et al., 2018), showing contrasting patterns.

Therefore, flooding-induced changes in plant secondary metabolites show variable but interpretable patterns. Several studies report increased production of defensive compounds, such as flavonoids and phenolics, under flooding stress, which may enhance plant resistance to certain chewing herbivores. In contrast, other studies have documented reductions in defensive metabolites or shifts in metabolic allocation that increase plant susceptibility. These contrasting outcomes suggest that the direction of flooding-induced metabolite responses depends strongly on plant species, flooding duration, and even on different plant parts of the same species.

##### 2.2.1.3 Consequences for chewing insect herbivores

A study on maize investigated not only how flooding affects metabolic pathways but also its relation to feeding by *Spodoptera frugiperda* (Block et al., 2020). In this study, flooding was reported to remodel the phenylpropanoid pathway, that led to increased accumulation of compounds like 4-coumaric and cinnamic acids that are precursors of anti- insect maysins, hence inhibiting growth of *Spodoptera frugiperda.* These findings were supported by a recent study of a maize mutant deficient in chalcone synthase, which exhibited lower flavonoid levels and greater caterpillar growth under flooding conditions than wild-type plants, confirming that flood-induced flavonoid accumulation enhances insect resistance (Gorman et al., 2025).

The studies mentioned above suggest that flooding stress alters plant metabolic pathways, resulting in changes in the production of specialized secondary metabolites with significant effects on insect herbivore growth and development. Emerging patterns from available studies suggest flooding-induced production of defensive secondary metabolites can be translated into increased plant resistance to insect herbivores. However, current knowledge remains limited, with most studies focused on a few compound classes and model plant species. Comprehensive metabolomic profiles across diverse crop species and wild relatives are lacking. Moreover, we are lacking in studies investigating how these documented flooding-plant-insect interactions impact change during the post-flood recovery phase and when flooding occurs simultaneously with other stressors. We are still missing the inclusions of herbivores representing different feeding guilds, dietary breadth specialization, different developmental stages, different densities of herbivores and different feeding durations. To fully understand the ecological and agricultural importance of these changes, it is crucial to expand research to encompass a broader range of secondary metabolites and plant systems, thereby revealing the full scope of flooding-induced shifts in plant defensive metabolites.

#### 2.2.2 Volatile organic compounds

Plant volatile organic compounds (VOCs) are some of the most chemically diverse secondary metabolites released from various parts of plants and facilitate both intra- and interspecific interactions (Turlings et al., 1998; War et al., 2011; Turlings and Erb, 2018; Kutty and Mishra, 2023). In addition to biotic stresses, such as insect herbivory, plants increase VOC emission by adjusting their production in response to abiotic stresses, including higher temperatures, elevated CO2 levels, drought, and flooding (Kesselmeier and Staudt, 1999; Brilli et al., 2019).

##### 2.2.2.1 Mechanisms of flooding-induced changes

The mechanisms behind flooding’s impact on VOCs production are complex, involving both direct and indirect effects. Flooding and hypoxia reduce gas exchange, forcing plants into anaerobic metabolism and redirecting precursors (e.g., from glycolysis) into stress-related secondary metabolism. This can change the amount and types of VOCs emitted. Additionally, flooding stress triggers hormonal crosstalk that modifies VOC biosynthesis pathways. For example, ethylene buildup influences VOC biosynthetic genes, particularly those involved to sesquiterpene biosynthesis, whereas flooding-induced JA and SA accumulation activate defense-related VOCs, such as GLVs, methyl jasmonate, and methyl salicylate (Pérez-Llorca et al., 2023). Flooding also alters phenylpropanoid metabolism (a precursor to benzenoids, coumarins, and flavonoids), often shifting emissions toward volatile defense compounds when combined with insect or pathogen attacks. In maize (*Z. mays*), under flooding and herbivory by *Spodoptera frugiperda*, an enhanced phenylpropanoid pathway led to increased production of volatile phenolic compounds, such as benzyl acetate and phenethyl acetate, in infested plants compared with controls (Block et al., 2020).

##### 2.2.2.2 Emerging patterns associated with this pathway

When comparing VOCs emissions from maize (*Z. mays*) and teosinte (*Z. nicaraguensis*) under flooding and insect herbivory (*Spodoptera frugiperda*), scientists observed that flooding stress alone increased VOCs emissions in both species, surpassing the levels caused by herbivory alone and the combined stress of flooding and herbivory. They further analyzed individual volatile compounds and identified 2-ethylcyclopentone, a ketone that contributes to the rise in VOC emissions due to flooding (Mleziva and Ngumbi, 2024). Similarly, Block et al. (2020) also found significantly higher emission of specific volatiles, homoterpene (3*E*, 7*E*)-4,8,12-trimethyl-1,3,7,11-tridecatetraene (TMTT) and the monoterpene alcohol linalool in *S. frugiperda*-infested maize plants that were flooded compared with nonflooded plants. Similar results were found in two maize hybrids, in which flooding and the combination of flooding and herbivory (*S. frugiperda*) drastically increased VOC production (Ngumbi and Ugarte, 2021). Beyond maize, the effect of flooding on VOC emissions has also been examined in tomato. For example, two heirloom varieties, Cherokee Purple and Striped German, were studied under flooding, herbivory (beet armyworm, *Spodoptera exigua*), and combined flooding and herbivory stress. Results showed that both individual flooding stress and the combined stress increased VOC emissions more than herbivory alone and no stress conditions. The analysis of individual compounds revealed that monoterpenes- α-terpinolene, (+)-4-carene, α-pinene, β-pinene, o-cymene and p-cymene, β-phellandrene; sesquiterpenes- humulene, δ-elemene and caryophyllene played significant roles in these differences (Ngumbi et al., 2022).

The emerging pattern from available studies suggests enhanced emissions of VOCs following flooding. However, our current understanding of how flooding affects their emission is limited to only a few plant species and cultivars. Empirical studies on the ecological relevance of these flooding induced VOCs emissions remain largely unexplored including influence on plant-insect interactions, such as attracting or repelling pest insects, affecting oviposition behavior, or attracting insect predators and parasitoids. Given the well-established role that VOCs play in plant- insect communication and multi-trophic interactions, future studies should investigate how flooding-induced changes in VOC emissions affect herbivores belonging to different feeding guilds as well as higher trophic levels. Additionally, the available flooding- VOCs related studies have been conducted in greenhouse settings, highlighting a strong need to expand research to field-based studies across multiple plant species and genotypes to better understand ecological consequences under natural conditions.

### 2.3 Pathway 3: Altering plant nutritional quality

Plant nutritional quality is key to plant-insect interactions, as it can directly and indirectly influence herbivore performance, population dynamics, and preferences (Behmer, 2009). Insects depend on plants for vital nutrients, including nitrogen, amino acids, sugars, proteins, and soluble carbohydrates. Changes in these resources can greatly affect insect growth, reproduction, and survival (Roeder and Behmer, 2014; Wetzel et al., 2016; Deans et al., 2022). Furthermore, plant nutritional quality interacts with plant defenses, influencing the tradeoffs between growth and defense (Bryant et al., 1983; Zheng et al., 2021). Therefore, understanding how plant nutritional quality varies under stress is essential for predicting insect population dynamics, responses, and the outcomes within agroecosystems. Plant nutritional quality is highly variable and depends greatly on environmental factors.

#### 2.3.1 Mechanisms of flooding-induced changes

Flooding impacts plant nutritional quality through various interconnected soil, root, and physiological processes, which affect nutrient content and balance in plant tissues (Soares et al., 2019). The anaerobic conditions in soil cause nutrient loss, especially nitrogen loss through denitrification, leaching, and surface runoff (Zurweller et al., 2015). Additionally, impaired root respiration reduces the ATP available for nutrient uptake and is exacerbated by root decay caused by soil toxicity from ions such as manganese, iron, and sulfide (Colmer and Voesenek, 2009; Lamers et al., 2013; Sasidharan et al., 2018). Furthermore, flooding reduces root branching and the number of fine roots, thereby limiting nutrient uptake from the soil (Vallino et al., 2014; Yamauchi et al., 2021). Limited oxygen during respiration forces plants to use anaerobic pathways to produce ATP, requiring high carbon input and potentially causing imbalances in carbohydrate levels, including glucose, fructose, and sucrose (Robertson et al., 2009).

#### 2.3.2 Emerging patterns associated with this pathway

Flooding is found to cause complex changes in plant macronutrient, micronutrient, and sugar content. For example, wheat (*Triticum aestivum*) plants under waterlogging stress were reported to have leaf nitrogen, potassium, and phosphorus levels, reduced by nearly half compared to non-stressed plants, due to decreased root-to-shoot nutrient translocation. Moreover, carbon was found to accumulate in the roots, indicating major disruptions in C and N metabolism (Cid et al., 2024). In *Medicago truncatula* under waterlogging, the nitrogen percentage was lower in the leaves but higher in the roots. Additionally, waterlogging induced complex changes in the sugar content of leaves and roots, primarily by inhibiting phloem translocation, resulting in higher sucrose concentrations and increased amino acid levels, as well as lower organic acid levels in shoot phloem sap (Lothier et al., 2020).

These drastic changes in phloem sap composition can directly affect populations of phloem-feeding insects and cause varied responses for generalist versus specialist herbivores. For example, in *Arabidopsis thaliana*, flooding reduced the performance of the generalist aphid *Myzus persicae* more than drought conditions, while the specialist *Brevicoryne brassicae* showed no change. Phloem analysis revealed that flooding (waterlogging) reduced amino acid and sucrose levels compared to drought, likely explaining the decreased performance of the generalist aphid (Mewis et al., 2012). A similar pattern was observed in *Rorippa palustris* plants, where aphid species such as *Myzus persicae* and *Lipaphis erysimi* exhibited impaired performance on waterlogged plants compared to control and drought-stressed plants, suggesting flooding induces plant resistance to phloem feeders (Kamps and Poelman, 2024).

These studies lay the foundation for understanding how flooding impacts the growth and population dynamics of insect herbivores across various feeding guilds and levels of specialization. Based on available studies, it is evident that the effects of flooding on the nutritional quality of plant leaves and phloem sap are complex. Since plant nutritional quality directly influences pest populations and can alter ecological interactions, there is an urgent need to expand these studies to include other genotypes and insect species.

### 2.4 Pathway 4: Plant changes during the post-flood recovery phase

While several physiological and ecological mechanisms have been proposed to explain post-flooding-mediated plant-insect interactions, these pathways have not been directly tested experimentally. Hypothetically, these mechanisms could impact insect herbivore growth and performance and represent promising hypotheses warranting further empirical investigation.

#### 2.4.1 Post-flooding associated plant stresses

Flooding is a sequential stress for plants, with the post-flood recovery phase including reoxygenation stress, dehydration, nutrient deficiencies, and disrupted soil microbial networks being as critical as the flooding event itself (Tamang and Fukao, 2015b; Yeung et al., 2019; Shikov et al., 2020; Vries et al., 2024).Notably, post- flooding plants show considerably higher activity of certain peroxidases and amino acids to facilitate faster recovery, which could change the physiological responses of post-flooded plants as compared to the flooded plants (Khan and Komatsu, 2016; Komatsu et al., 2024). These cascading effects of flooding could deplete plant resources and weaken defenses, making plants highly vulnerable to environmental stresses, including insect herbivory, with major implications for insect growth and performance (Yeung et al., 2019; Yuan et al., 2023).

Although the details are still not fully explored, existing evidence indicates several ways this phase could potentially impact plant-insect interactions. Plants’ roots recover slowly after floodwaters recede, which hampers their ability to absorb nutrients, leading to nutritional imbalances that can significantly affect insect herbivores (Robertson et al., 2009). The surge of reactive oxygen species (ROS) caused by sudden reoxygenation can divert the plant’s resources from defense-related compounds to ROS-scavenging enzymes and antioxidants, temporarily making plants more vulnerable to insect herbivores (Yeung et al., 2018). Additionally, plant hormones such as jasmonic acid, ethylene, and ABA are known to be vital during the reoxygenation phase (León et al., 2021). For instance, in Arabidopsis, a rapid increase in jasmonates and higher transcript levels of JA biosynthesis genes have been *observed* during reoxygenation (Yuan et al., 2017). Along with this, in several *Rumex* spp., post flooding ethylene was found be increased 10-fold as compared to flooding (Voesenek et al., 2003). Since these are key hormones in plant defense against insects, this surge induced by reoxygenation can have notable effects on insect herbivores. Furthermore, reoxygenation often accelerates leaf senescence, leading to increased necrotic tissue, which can alter the palatability and feeding preferences of insect herbivores, particularly leaf-chewing species (Yeung et al., 2019).

Finally, the post-flood recovery phase alters plant physiology, defense signaling, and nutritional balance, creating an ecologically important window in which herbivores can exploit plant weakness, potentially reshaping pest outbreaks. Ignoring the recovery phase and focusing solely on the flooding period provides an incomplete picture, often underestimating the impact of flooding on the dynamics of plant-insect interactions.

#### 2.4.2 Grower’s Interventions - Nitrogen Application

Flooding greatly reduces soil nitrate levels through several processes, including surface runoff, leaching, and particularly denitrification by anaerobic denitrifying bacteria (Zurweller et al., 2015; Kaur et al., 2020) and by limiting nitrogen uptake sue to impaired root conductance (Robertson et al., 2009). Because of these losses, additional nitrogen fertilization after flooding has been shown to help plants recover and increase yields (Nielsen, 2015; Novais et al., 2025). Applying nitrogen is reported to improve plant tolerance to flooding stress by enhancing adaptive mechanisms, such as forming adventitious roots, which can lead to higher crop yields (Jaiswal and Srivastava, 2015; Kaur et al., 2017, Kaur et al., 2020), indicating that growers should think about applying nitrogen after flood events (Wu et al., 2014; Kaur et al., 2017).

Although the effects of after-flood nitrogen application on insect herbivores have not been experimentally tested, additional nitrogen application can cause various changes in plant traits, such as changing nutritional quality, that influence plant-insect interactions. Additionally, as predicted by carbon-nutrient balance hypothesis, higher nitrogen availability can reduce the production of carbon-based secondary metabolites, such as phenolics and tannins, as plants allocate more resources to growth rather than to defense (Bryant et al., 1983). Moreover, nitrogen fertilization can boost plant growth and biomass, which may attract more insect herbivores and eventually lead to increased pest outbreaks (Price, 1991). Insects such as aphids, whiteflies, and leafhoppers feed on plant phloem sap by piercing the phloem sieve elements using their stylet-like mouthparts (Dixon, 1997). The main components of phloem sap are sucrose and amino acids, whose concentrations can vary dramatically in response to many biotic and abiotic factors (Karley et al., 2002). N fertilizers’-induced changes in phloem sap can influence insect herbivore’s development and fecundity in a complicated manner. However, whether nitrogen applied after flooding produces similar effects on insect herbivores remains unknown and represents an important area for future research.

Therefore, nitrogen fertilization can significantly influence plant nutritional quality, secondary metabolism, and consequently plant–insect interactions. However, little is known about how these dynamics develop when nitrogen is applied after flooding stress. Flooding significantly affects soil nitrogen availability and plant uptake; however, we lack studies examining how post-flood nitrogen recovery interacts with plant defense pathways, metabolism, and ultimately, susceptibility or resistance to insect herbivores.

## 3 Synthesis statement from all pathways

Collectively, flooding is a multidimensional and integrative stressor that simultaneously reprograms and changes plants’ metabolic, hormonal, nutritional, and ecological traits, and across studies, several consistent patterns emerged. Flooding increases secondary metabolites, mainly phenolics, flavonoids, enhances VOC emissions, and reduces plant nutritional quality, such as Nitrogen. These flooding-induced changes and pathways can act in isolation or interactively. Therefore, flooding can reshape plant-insect interactions across multiple ecological scales, from affecting individual herbivore growth, performance, and behavior to altering community composition and trophic dynamics. These ecological outcomes are further shaped by herbivore identity, feeding guild and dietary breadth, herbivore density, whether flooding is occurring in isolation or with other stressors, and plant evolutionary and breeding history.

## 4 Limitations of our current knowledge

Despite the growing recognition that flooding, an ecologically important abiotic stressor, is increasing in frequency and intensity, major limitations remain in our understanding of how flooding shapes plant-insect interactions. As discussed in this review, existing studies provide emerging evidence that flooding significantly affects plant traits involved in plant-insect interactions. The actual impact of flooding on these interactions is probably even greater than what has been reported. However, these studies have several limitations that warrant further investigation. For example, they mainly focus on a few model species and genotypes, such as maize and tomato.

Current flooding studies lack exploration into specialized secondary metabolites. Many secondary metabolites are essential for the plant’s response to flooding and also affect plant-insect interactions. For example, maize produces specialized metabolites called, which are reported to help defend the plant against biotic stresses, particularly insect herbivory, and play roles in abiotic stress mitigation, including oxidative stress that accompanies flooding (Zhou et al., 2018). Our understanding of how flooding influences the specialized metabolites of key crops is limited and needs further research.

In addition, plants produce specialized signaling and defensive volatile compounds such as terpenoids. Although studies have begun to examine the influence of flooding on these volatiles’ production (**Table 1**), the ecological relevance of such changes, particularly in terms of their effects on herbivore oviposition or natural enemies attraction remains largely unexplored. Moreover, several studies have tested the effects of flooding on insect herbivores’ growth and performance, but they have not considered the broader picture and have stopped at insect growth, without testing the plant responses that drive that particular outcome.

Regarding insect herbivores, only a few insect species have been tested so far for flooding studies. Insects from different feeding guilds elicit distinct plant responses based on their oral secretions and feeding patterns (Poelman and Dicke, 2014). For example, leaf-chewing insects disturb plants while feeding and trigger changes associated with the Jasmonic acid signaling pathway, whereas sap-feeding insects cause cell damage by inserting their stylets into plant cells to reach the phloem, inducing changes associated with the Salicylic acid signaling pathway (Moreira et al., 2018). Considering the crosstalk between phytohormones and the complications that arise from it, it is highly unexplored how flooding relates to insect herbivores from various feeding guilds and levels of specialization.

Most research on flooding and plant–insect interactions overlook the critical post-flood recovery phase, during which plants are highly vulnerable, offering insects opportunities to exploit weakened hosts for feeding and oviposition.

Finally, existing flooding studies on insect herbivory have been conducted in greenhouse settings. Most of the greenhouse study results have not been validated in field conditions.

## 5 Future Directions

The current lack of empirical data is one of the biggest hurdles in our understanding of flooding-plant-insect interactions. Here we suggest some areas of research that could help elucidate the multi-pathways through which flooding influence the dynamics of plant- insect interactions.

- Expand research to diverse crop species such as soybean (*Glycine max*), rice (*Oryza sativa*), and wheat (*Triticum aestivum*) that are highly susceptible to flooding and experience substantial yield losses (Kim et al., 2023).
- Include insect herbivores belonging to diverse feeding guilds and different levels of specializations in flooding-related studies.
- Test how flooding influences the production of specialized metabolites, for instance benzoxazinoids in maize.
- Test the ecological relevance of the changed emission of certain specialized VOCs, particularly in terms of their effects on insect herbivore oviposition or natural enemy attraction.
- Test the holistic picture of flooding-plant and insect interactions by including both insect growth outcome along with the plant response that drives that insect outcome.
- Flooding-related experimental designs must include the recovery phase to obtain a complete picture. This phase accompanies its own stresses for plants, such as reoxygenation, nutrient deficiencies, and may reprogram the pathways as compared to the flooding phase.
- Test the influence of nitrogen application in varying doses and in different forms to get a complete picture. Nitrogen fertilization after flooding is a vital strategy to support plant stress responses. Clarifying the role of N supplementation following flooding in restoring plant defenses or improving insect performance is essential.
- Combine greenhouse studies with field-based studies to verify the outcomes.

## 6 Conclusions

The increasing severity and intensity of flooding as a global concern necessitate research into the sequential impacts of flooding stress on plant traits and the ecological effects on insect herbivores. Clearly, flooding-plant and insect interactions are complex and multifaceted, making generalizations difficult. Evidence from existing studies suggests that flooding does not produce uniform effects on herbivores. In some systems, flooding suppresses jasmonate-mediated defenses and increases herbivore performance, particularly for sap-feeding insects. In other cases, flooding can induce defensive pathways or alter plant chemistry in ways that reduce herbivore growth, particularly for chewing insect herbivores. These contrasting outcomes indicate that flooding may both support and modify predictions of existing plant ecological theories (such as plant stress and plant vigor hypotheses), depending on plant species, herbivore feeding guild, and flooding intensity or duration (**Table 1**). Evidently, flooding stress is unique and differs mechanistically from other abiotic plant stresses, such as drought, whose impacts on insect herbivory have been widely generalized and used to develop the frameworks of many ecological theories (English-Loeb et al., 1997; Tariq et al., 2012; Grinnan et al., 2013; Weldegergis et al., 2015). Moreover, the mechanistic pathways presented in this review are discussed separately for clarity; they are often interconnected. For instance, phytohormone signaling can regulate the biosynthesis of secondary metabolites and VOCs, which in turn influence herbivore behavior and plant defense structure. Nevertheless, existing knowledge on flooding-plant-insect interactions presents several testable predictions that could substantially advance our understanding of this field. For example, flooding-induced metabolic reprogramming may alter the levels of defensive compounds such as flavonoids, thereby affecting insect growth and performance. Similarly, increased emission of flooding-induced VOCs will affect insect host selection or natural enemy attraction, to name a few. Addressing these predictions will require multidisciplinary approaches that combine metabolomics, transcriptomics, and ecological assays, which are urgently needed to create predictive models for flooding-induced changes in plant–insect interactions. Ultimately, our identified patterns highlight flooding a significant abiotic stressor to consider when studying plant-insect relationships. Outcomes and results from the studies that put flooding at the center of abiotic stress-plant insect interactions studies could facilitate the refining of long-standing ecological frameworks and theories. Therefore, flooding represents a unique and novel stressor that offers a powerful lens and an opportunity to advance our understanding of ecological theories and hence plant- insect ecology.

## References

[B1] Adegoye G. A. Olorunwa O. J. Alsajri F. A. Walne C. H. Wijewandana C. Kethireddy S. R. . (2023). Waterlogging effects on soybean physiology and hyperspectral reflectance during the reproductive stage. Agriculture 13, 844. doi: 10.3390/agriculture13040844. PMID: 41725453

[B2] Akula R. Ravishankar G. A. (2011). Influence of abiotic stress signals on secondary metabolites in plants. Plant Signaling Behav. 6, 1720–1731. doi: 10.4161/psb.6.11.17613. PMID: 22041989 PMC3329344

[B3] Altieri M. A. Nicholls C. I. . (1999). Biodiversity, ecosystem function and insect pest management in agricultural systems. In Biodiversity in Agroecosystems. Collins W. W. Qualset C. O. (Eds.). (Boca Raton: CRC Press). Available online: https://dlib.scu.ac.ir/bitstream/Hannan/371524/2/1566702909.pdf#page=75.

[B4] Bailey-Serres J. Fukao T. Gibbs D. J. Holdsworth M. J. Lee S. C. Licausi F. . (2012). Making sense of low oxygen sensing. Trends Plant Sci. 17, 129–138. doi: 10.1016/j.tplants.2011.12.004. PMID: 22280796

[B5] Bauerfeind S. S. Fischer K. (2013). Increased temperature reduces herbivore host-plant quality. Global Change Biology 19, 3272–3282. doi: 10.1111/gcb.12297, PMID: 23775632

[B6] Behmer S. T. (2009). Insect herbivore nutrient regulation. Annu. Rev. Entomol. 54, 165–187. doi: 10.1146/annurev.ento.54.110807.090537. PMID: 18764740

[B7] Bennett R. N. Wallsgrove R. M. (1994). Secondary metabolites in plant defence mechanisms. New Phytol. 127, 617–633. doi: 10.1111/j.1469-8137.1994.tb02968.x. PMID: 33874382

[B8] Block A. K. Hunter C. T. Sattler S. E. Rering C. McDonald S. Basset G. J. . (2020). Fighting on two fronts: Elevated insect resistance in flooded maize. Plant, Cell & Environment 43, 223–234. doi: 10.1111/pce.13642, PMID: 31411732

[B9] Brilli F. Loreto F. Baccelli I. (2019). Exploiting plant volatile organic compounds (VOCs) in agriculture to improve sustainable defense strategies and productivity of crops. Front. Plant Sci. 10. doi: 10.3389/fpls.2019.00264. PMID: 30941152 PMC6434774

[B10] Bryant J. P. Chapin F. S. Klein D. R. (1983). Carbon/nutrient balance of boreal plants in relation to vertebrate herbivory. Oikos 40, 357–368. doi: 10.2307/3544308. PMID: 39964225

[B11] Cid G. A. Francioli D. Kolb S. Tandron Moya Y. A. von Wirén N. Hajirezaei M.-R. (2024). Transcriptomic and metabolomic approaches elucidate the systemic response of wheat plants under waterlogging. J. Exp. Bot. 75, 1510–1529. doi: 10.1093/jxb/erad453. PMID: 38014629

[B12] Colmer T. D. Voesenek L. A. C. J. (2009). Flooding tolerance: suites of plant traits in variable environments. Funct. Plant Biol. 36, 665. doi: 10.1071/FP09144. PMID: 32688679

[B13] Coutinho I. D. Henning L. M. M. Döpp S. A. Nepomuceno A. Moraes L. A. C. Marcolino-Gomes J. . (2018). Flooded soybean metabolomic analysis reveals important primary and secondary metabolites involved in the hypoxia stress response and tolerance. Environ. Exp. Bot. 153, 176–187. doi: 10.1016/j.envexpbot.2018.05.018. PMID: 41936479

[B14] Deans C. A. Sword G. A. Vogel H. Behmer S. T. (2022). Quantity versus quality: Effects of diet protein-carbohydrate ratios and amounts on insect herbivore gene expression. Insect Biochem. Mol. Biol. 145, 103773. doi: 10.1016/j.ibmb.2022.103773. PMID: 35405259

[B15] DeLucia E. H. Nabity P. D. Zavala J. A. Berenbaum M. R. (2012). Climate Change: Resetting Plant-Insect Interactions. Plant Physiology 160, 1677–1685. doi: 10.1104/pp.112.204750, PMID: 22972704 PMC3510101

[B16] De Ollas C. González-Guzmán M. Pitarch Z. Matus J. T. Candela H. Rambla J. L. . (2021). Identification of ABA-mediated genetic and metabolic responses to soil flooding in tomato (Solanum lycopersicum L. Mill). Front. Plant Sci. 12. doi: 10.3389/fpls.2021.613059. PMID: 33746996 PMC7973378

[B17] Dixon A. F. G. (1997). Aphid Ecology An optimization approach (London, UK: Springer Science & Business Media).

[B18] Dofuor A. K. Osei-Owusu J. Osabutey A. F. Lutuf H. Antwi-Agyakwa A. K. Andoh-Mensah S. . (2024). Plant-insect interactions under agroecosystems: an overview of ecological implications for future research. Cogent. Food. Agric. 10, 2379606. doi: 10.1080/23311932.2024.2379606. PMID: 41909888

[B19] Dorschner K. W. Johnson R. C. Eikenbary R. D. Ryan J. D. (1986). Insect-plant interactions: Greenbugs (Homoptera: Aphididae) disrupt acclimation of winter wheat to drought stress. Environ. Entomol. 15, 118–121. doi: 10.1093/ee/15.1.118. PMID: 34916285

[B20] EL Sabagh A. Islam M. S. Hossain A. Iqbal M. A. Mubeen M. Waleed M. . (2022). Phytohormones as growth regulators during abiotic stress tolerance in plants. Front. Agron. 4. doi: 10.3389/fagro.2022.765068. PMID: 41930257

[B21] English-Loeb G. Stout M. J. Duffey S. S. (1997). Drought stress in tomatoes: Changes in plant chemistry and potential nonlinear consequences for insect herbivores. Oikos 79, 456–468. doi: 10.2307/3546888. PMID: 39964225

[B22] Fauvel G. (1999). Diversity of Heteroptera in agroecosystems: role of sustainability and bioindication. In Invertebrate Biodiversity as Bioindicators of Sustainable Landscapes. Paoletti M. G. (Ed.). (Amsterdam: Elsevier), 275–303. doi: 10.1016/B978-0-444-50019-9.50016-9, PMID:

[B23] Fraenkel G. (1969). Evaluation of our thoughts on secondary plant substances. Entomol. Exp. Appl. 12, 473–486. doi: 10.1111/j.1570-7458.1969.tb02546.x. PMID: 41940437

[B24] Fukao T. Barrera-Figueroa B. E. Juntawong P. Peña-Castro J. M. (2019). Submergence and waterlogging stress in plants: A review highlighting research opportunities and understudied aspects. Front. Plant Sci. 10. doi: 10.3389/fpls.2019.00340. PMID: 30967888 PMC6439527

[B25] Gao S. Wang Y. Yu S. Huang Y. Liu H. Chen W. . (2020). Effects of drought stress on growth, physiology and secondary metabolites of two Adonis species in Northeast China. Sci. Hortic. 259, 108795. doi: 10.1016/j.scienta.2019.108795. PMID: 41936479

[B26] Gely C. Laurance S. G. W. Stork N. E. (2020). How do herbivorous insects respond to drought stress in trees? Biol. Rev. 95, 434–448. doi: 10.1111/brv.12571. PMID: 31750622

[B27] Giron D. Dubreuil G. Bennett A. Dedeine F. Dicke M. Dyer L. A. . (2018). Promises and challenges in insect–plant interactions. Entomologia Experimentalis et Applicata 166, 319–343. doi: 10.1111/eea.12679, PMID: 40046247

[B28] Gorman Z. Liu H. Sorg A. Grissett K. S. Yactayo-Chang J. P. Li Q.-B. . (2025). Flood-induced insect resistance in maize involves flavonoid-dependent salicylic acid induction. Plant Cell Environ. 48, 5169–5183. doi: 10.1111/pce.15496. PMID: 40162687

[B29] Grinnan R. Carter T. E. Johnson M. T. J. (2013). Effects of drought, temperature, herbivory, and genotype on plant–insect interactions in soybean (Glycine max). Arthropod-Plant. Interact. 7, 201–215. doi: 10.1007/s11829-012-9234-z. PMID: 41933263

[B30] Hamann E. Blevins C. Franks S. J. Jameel M. I. Anderson J. T. (2021). Climate change alters plant–herbivore interactions. New Phytologist 229, 1894–1910. doi: 10.1111/nph.17036, PMID: 33111316

[B31] Han C. Dong J. Zhang G. Zhu Q. Yu F. (2025). Root ethylene and abscisic acid responses to flooding stress in Styrax japonicus: A transcriptomic perspective. Plants 14, 1870. doi: 10.3390/plants14121870. PMID: 40573857 PMC12197084

[B32] Hettenhausen C. Schuman M. C. Wu J. (2015). MAPK signaling: A key element in plant defense response to insects. Insect Sci. 22, 157–164. doi: 10.1111/1744-7917.12128. PMID: 24753304 PMC5295641

[B33] Howe G. A. Lightner J. Browse J. Ryan C. A. (1996). An octadecanoid pathway mutant (JL5) of tomato is compromised in signaling for defense against insect attack. Plant Cell 8, 2067–2077. doi: 10.1105/tpc.8.11.2067. PMID: 8953771 PMC161335

[B34] Huberty A. F. Denno R. F. (2004). Plant Water Stress and Its Consequences for Herbivorous Insects: A New Synthesis. Ecology 85, 1383–1398. doi: 10.1890/03-0352

[B35] Jaiswal A. Srivastava J. P. (2015). Effect of nitric oxide on some morphological and physiological parameters in maize exposed to waterlogging stress. African Journal of Agricultural Research 10, 3462–3471. doi: 10.5897/AJAR2015.9790

[B36] Jankielsohn A. (2018). The Importance of Insects in Agricultural Ecosystems. Advances in Entomology 6, 62–73. doi: 10.4236/ae.2018.62006

[B37] Kaji M. Katano K. Anee T. I. Nitta H. Yamaji R. Shimizu R. . (2024). Response of Arabidopsis thaliana to flooding with physical flow. Plants 13, 3508. doi: 10.3390/plants13243508. PMID: 39771206 PMC11678080

[B38] Kamps B. B. J. Poelman E. H. (2024). Adaptations to water gradient in three Rorippa plant species correspond with plant resistance against insect herbivory under drought and waterlogged conditions. Ecol. Entomol. 49, 1–9. doi: 10.1111/een.13273. PMID: 41940437

[B39] Kansman J. T. Basu S. Casteel C. L. Crowder D. W. Lee B. W. Nihranz C. T. . (2022). Plant Water Stress Reduces Aphid Performance: Exploring Mechanisms Driven by Water Stress Intensity. Frontiers in Ecology and Evolution 10. doi: 10.3389/fevo.2022.846908

[B40] Karley A. J. Douglas A. E. Parker W. E. (2002). Amino acid composition and nutritional quality of potato leaf phloem sap for aphids. J. Exp. Biol. 205, 3009–3018. doi: 10.1242/jeb.205.19.3009. PMID: 12200404

[B41] Kaur G. Singh G. Motavalli P. P. Nelson K. A. Orlowski J. M. Golden B. R. (2020). Impacts and management strategies for crop production in waterlogged or flooded soils: A review. Agron. J. 112, 1475–1501. doi: 10.1002/agj2.20093. PMID: 41925065

[B42] Kaur G. Zurweller B. A. Nelson K. A. Motavalli P. P. Dudenhoeffer C. J. (2017). Soil waterlogging and nitrogen fertilizer management effects on corn and soybean yields. Agron. J. 109, 97–106. doi: 10.2134/agronj2016.07.0411

[B43] Kesselmeier J. Staudt M. (1999). Biogenic volatile organic compounds (VOC): An overview on emission, physiology and ecology. J. Atmos. Chem. 33, 23–88. doi: 10.1023/A:1006127516791. PMID: 41886696

[B44] Khan M. N. Komatsu S. (2016). Characterization of post-flooding recovery-responsive enzymes in soybean root and hypocotyl. J. Plant Biol. 59, 478–487. doi: 10.1007/s12374-016-0048-x. PMID: 41933263

[B45] Khan M. I. R. Trivellini A. Chhillar H. Chopra P. Ferrante A. Khan N. A. . (2020). The significance and functions of ethylene in flooding stress tolerance in plants. Environ. Exp. Bot. 179, 104188. doi: 10.1016/j.envexpbot.2020.104188. PMID: 41936479

[B46] Kim W. Iizumi T. Hosokawa N. Tanoue M. Hirabayashi Y. (2023). Flood impacts on global crop production: Advances and limitations. Environ. Res. Lett. 18, 054007. doi: 10.1088/1748-9326/accd85

[B47] Komatsu S. Egishi M. Ohno T. (2024). The changes of amino-acid metabolism between wheat and rice during early growth under flooding stress. Int. J. Mol. Sci. 25, 5229. doi: 10.3390/ijms25105229. PMID: 38791268 PMC11121113

[B48] Kumar S. Bhushan B. Wakchaure G. C. Meena K. K. Kumar M. Meena N. L. . (2020). “ Plant phenolics under water-deficit conditions: Biosynthesis, accumulation, and physiological roles in water stress alleviation,” in Plant Phenolics in Sustainable Agriculture: Volume 1. Eds. Lone R. Shuab R. Kamili A. N. ( Springer, Singapore), 451–465. doi: 10.1007/978-981-15-4890-1_19

[B49] Kumar K. Debnath P. Singh S. Kumar N. (2023). An overview of plant phenolics and their involvement in abiotic stress tolerance. Stresses 3, 570–585. doi: 10.3390/stresses3030040. PMID: 41725453

[B50] Kutty N. N. Mishra M. (2023). Dynamic distress calls: Volatile info chemicals induce and regulate defense responses during herbivory. Front. Plant Sci. 14. doi: 10.3389/fpls.2023.1135000. PMID: 37416879 PMC10322200

[B51] Lamers L. P. M. Govers L. L. Janssen I. C. Geurts J. J. Van der Welle M. E. Van Katwijk M. M. . (2013). Sulfide as a soil phytotoxin—a review. Front. Plant Sci. 4. doi: 10.3389/fpls.2013.00268. PMID: 23885259 PMC3717504

[B52] Lee H.-J. Park J.-S. Shin S. Y. Kim S.-G. Lee G. Kim H.-S. . (2020). Submergence deactivates wound-induced plant defence against herbivores. Commun. Biol. 3, 1–9. doi: 10.1038/s42003-020-01376-4. PMID: 33159149 PMC7648080

[B53] León J. Castillo M. C. Gayubas B. (2021). The hypoxia–reoxygenation stress in plants. J. Exp. Bot. 72, 5841–5856. doi: 10.1093/jxb/eraa591. PMID: 33367851 PMC8355755

[B54] Lewis M. T. Poelstra J. W. Michel A. P. (2025). Host plant flooding stress in soybeans differentially impacts avirulent and virulent soybean aphid (Aphis glycines) biotypes. Sci. Rep. 15, 4897. doi: 10.1038/s41598-025-87561-z. PMID: 39929874 PMC11811272

[B55] Li H. Huang L. Tang L. Liu J. Zhang P. Wang X. . (2026). Integrated physiology, transcriptomics and metabolomics analysis of the effects of flooding stress on the accumulation of flavonoids and organic acids in quinoa seeds. J. Sci. Food Agric. 106, 150–171. doi: 10.1002/jsfa.70152. PMID: 40955649

[B56] Lothier J. Diab H. Cukier C. Limami A. M. Tcherkez G. (2020). Metabolic responses to waterlogging differ between roots and shoots and reflect phloem transport alteration in Medicago truncatula. Plants 9, 1373. doi: 10.3390/plants9101373. PMID: 33076529 PMC7650564

[B57] Manik S. M. N. Pengilley G. Dean G. Field B. Shabala S. Zhou M. (2019). Soil and crop management practices to minimize the impact of waterlogging on crop productivity. Front. Plant Sci. 10. doi: 10.3389/fpls.2019.00140. PMID: 30809241 PMC6379354

[B58] Mattson W. J. Haack R. A. . (1987). The Role of Drought in Outbreaks of Plant-Eating Insects. BioScience 37, 110–118. doi: 10.2307/1310365

[B59] Mewis I. Khan M. A. M. Glawischnig E. Schreiner M. Ulrichs C. (2012). Water stress and aphid feeding differentially influence metabolite composition in Arabidopsis thaliana (L.). PloS One 7, e48661. doi: 10.1371/journal.pone.0048661. PMID: 23144921 PMC3492492

[B60] Mhlongo M. I. Piater L. A. Steenkamp P. A. Labuschagne N. Dubery I. A. (2020). Metabolic profiling of PGPR-treated tomato plants reveal priming-related adaptations of secondary metabolites and aromatic amino acids. Metabolites 10, 210. doi: 10.3390/metabo10050210. PMID: 32443694 PMC7281251

[B61] Mleziva A. D. Ngumbi E. N. (2024). Comparative analysis of defensive secondary metabolites in wild teosinte and cultivated maize under flooding and herbivory stress. Physiologia Plantarum 176, e14216. doi: 10.1111/ppl.14216, PMID: 38366721

[B62] Moreira X. Abdala-Roberts L. Castagneyrol B. (2018). Interactions between plant defence signalling pathways: Evidence from bioassays with insect herbivores and plant pathogens. J. Ecol. 106, 2353–2364. doi: 10.1111/1365-2745.12987. PMID: 41940437

[B63] Mustroph A. (2018). Improving Flooding Tolerance of Crop Plants. Agronomy 8, 160. doi: 10.3390/agronomy8090160, PMID: 30654563

[B64] Ngumbi E. N. (2025). Could flooding undermine progress in building climate-resilient crops? Trends Plant Sci. 30, 85–94. doi: 10.1016/j.tplants.2024.07.017. PMID: 39168786

[B65] Ngumbi E. N. Ugarte C. M. (2021). Flooding and herbivory interact to alter volatile organic compound emissions in two maize hybrids. J. Chem. Ecol. 47, 707–718. doi: 10.1007/s10886-021-01286-7. PMID: 34125370

[B66] Ngumbi E. Dady E. Calla B. (2022). Flooding and herbivory: the effect of concurrent stress factors on plant volatile emissions and gene expression in two heirloom tomato varieties. BMC Plant Biology 22, 536. doi: 10.1186/s12870-022-03911-3, PMID: 36396998 PMC9670554

[B67] Nguyen D. D’Agostino N. Tytgat T. O. G. Sun P. Lortzing T. Visser E. J. W. . (2016). Drought and flooding have distinct effects on herbivore-induced responses and resistance in Solanum dulcamara. Plant Cell Environ. 39, 1485–1499. doi: 10.1111/pce.12708. PMID: 26759219

[B68] Nielsen R. L. (2015). Effects of flooding or ponding on corn prior to tasseling. Corny. News Network. Purdue. Univ.

[B69] Novais W. Sprunger C. D. Mann M. Lindsey L. E. Ortez O. A. Lindsey A. J. (2025). Assessing pre-plant nitrogen sources and waterlogging on corn growth and yield. Crop. Forage. Turfgrass Manage. 11, e70071. doi: 10.1002/cft2.70071. PMID: 41925065

[B70] Pérez-Llorca M. Pollmann S. Müller M. (2023). Ethylene and jasmonates signaling network mediating secondary metabolites under abiotic stress. Int. J. Mol. Sci. 24, 5990. doi: 10.3390/ijms24065990. PMID: 36983071 PMC10051637

[B71] Pincebourde S. van Baaren J. Rasmann S. Rasmont P. Rodet G. Martinet B. . (2017). “ Chapter nine - Plant–insect interactions in a changing world,” in Advances in Botanical Research. Eds. Sauvion N. Thiéry D. Calatayud P.-A. (London, UK: Academic Press), 289–332. doi: 10.1016/bs.abr.2016.09.009, PMID:

[B72] Poelman E. H. Dicke M. (2014). “ Plant-mediated interactions among insects within a community ecological perspective,” in Annual Plant Reviews (Chichester, UK: John Wiley & Sons, Ltd), 309–337. doi: 10.1002/9781118829783.ch9, PMID:

[B73] Price P. W. (1991). The plant vigor hypothesis and herbivore attack. Oikos 62, 244–251. doi: 10.2307/3545270. PMID: 39964225

[B74] Robertson D. Zhang H. Palta J. A. Colmer T. Turner N. C. (2009). Waterlogging affects the growth, development of tillers, and yield of wheat through a severe, but transient, N deficiency. Crop Pasture Sci. 60, 578. doi: 10.1071/CP08440. PMID: 41161682

[B75] Roeder K. A. Behmer S. T. (2014). Lifetime consequences of food protein-carbohydrate content for an insect herbivore. Funct. Ecol. 28, 1135–1143. doi: 10.1111/1365-2435.12262. PMID: 41940437

[B76] Rouault G. Candau J.-N. Lieutier F. Nageleisen L.-M. Martin J.-C. Warzée N. (2006). Effects of drought and heat on forest insect populations in relation to the 2003 drought in Western Europe. Ann. For. Sci. 63, 613–624. doi: 10.1051/forest:2006044

[B77] Sasidharan R. Bailey-Serres J. Ashikari M. Atwell B. J. Colmer T. D. Fagerstedt K. . (2017). Community recommendations on terminology and procedures used in flooding and low oxygen stress research. New Phytol. 214, 1403–1407. doi: 10.1111/nph.14519. PMID: 28277605

[B78] Sasidharan R. Hartman S. Liu Z. Martopawiro S. Sajeev N. van Veen H. . (2018). Signal dynamics and interactions during flooding stress. Plant Physiol. 176, 1106–1117. doi: 10.1104/pp.17.01232. PMID: 29097391 PMC5813540

[B79] Shikov A. E. Chirkova T. V. Yemelyanov V. V. (2020). Post-anoxia in plants: Reasons, consequences, and possible mechanisms. Russ. J. Plant Physiol. 67, 45–59. doi: 10.1134/S1021443720010203. PMID: 41411595

[B80] Singh S. Kaur I. Kariyat R. (2021). The multifunctional roles of polyphenols in plant-herbivore interactions. Int. J. Mol. Sci. 22, 1442. doi: 10.3390/ijms22031442. PMID: 33535511 PMC7867105

[B81] Soares J. C. Santos C. S. Carvalho S. M. P. Pintado M. M. Vasconcelos M. W. (2019). Preserving the nutritional quality of crop plants under a changing climate: Importance and strategies. Plant Soil 443, 1–26. doi: 10.1007/s11104-019-04229-0. PMID: 41933263

[B82] Stout M. J. Rita Riggio M. Zou L. Roberts R. (2002). Flooding influences ovipositional and feeding behavior of the rice water weevil (Coleoptera: Curculionidae). J. Econ. Entomol. 95, 715–721. doi: 10.1603/0022-0493-95.4.715. PMID: 12216811

[B83] Tamang B. G. Fukao T. (2015). Plant adaptation to multiple stresses during submergence and following desubmergence. Int. J. Mol. Sci. 16, 30164–30180. doi: 10.3390/ijms161226226. PMID: 26694376 PMC4691168

[B84] Tariq M. Wright D. J. Rossiter J. T. Staley J. T. (2012). Aphids in a changing world: Testing the plant stress, plant vigour and pulsed stress hypotheses. Agric. For. Entomol. 14, 177–185. doi: 10.1111/j.1461-9563.2011.00557.x. PMID: 41940437

[B85] Tindall K. V. Bernhardt J. L. Stout M. J. Beighley D. H. (2013). Effect of depth of flooding on the rice water weevil, Lissorhoptrus oryzophilus, and yield of rice. J. Insect Sci. 13, 1–9. doi: 10.1673/031.013.6201. PMID: 23906324 PMC3740922

[B86] Turlings T. C. J. Erb M. (2018). Tritrophic Interactions Mediated by Herbivore-Induced Plant Volatiles: Mechanisms, Ecological Relevance, and Application Potential. Annual Review of Entomology 63, 433–452. doi: 10.1146/annurev-ento-020117-043507, PMID: 29324043

[B87] Turlings T. C. J. Bernasconi M. Bertossa R. Bigler F. Caloz G. Dorn S. (1998). The induction of volatile emissions in maize by three herbivore species with different feeding habits: Possible consequences for their natural enemies. Biol. Control. 11, 122–129. doi: 10.1006/bcon.1997.0591. PMID: 39885891

[B88] Tylianakis J. M. Didham R. K. Bascompte J. Wardle D. A. (2008). Global change and species interactions in terrestrial ecosystems. Ecology Letters 11, 1351–1363. doi: 10.1111/j.1461-0248.2008.01250.x, PMID: 19062363

[B89] Umićević S. Kukavica B. Maksimović I. Gašić U. Milutinović M. Antić M. . (2024). Stress response in tomato as influenced by repeated waterlogging. Front. Plant Sci. 15. doi: 10.3389/fpls.2024.1331281. PMID: 39109057 PMC11300220

[B90] Vallino M. Fiorilli V. Bonfante P. (2014). Rice flooding negatively impacts root branching and arbuscular mycorrhizal colonization, but not fungal viability. Plant Cell Environ. 37, 557–572. doi: 10.1111/pce.12177. PMID: 23927052

[B91] Voesenek L. C. J. Jackson M. B. Toebes A. H. W. Huibers W. Vriezen W. H. Colmer T. D. (2003). De-submergence-induced ethylene production in Rumex palustris: Regulation and ecophysiological significance. Plant J. 33, 341–352. doi: 10.1046/j.1365-313X.2003.01632.x. PMID: 12535347

[B92] Voesenek L. C. J. Sasidharan R. (2013). Ethylene – and oxygen signalling – drive plant survival during flooding. Plant Biol. 15, 426–435. doi: 10.1111/plb.12014. PMID: 23574304

[B93] Vries M. C. J. Kruijf J. V. Augustijn D. C. M. (2024). Towards improved flood resilience: Integrating flood protection in spatial planning in urbanized deltas. Available online at: https://research.utwente.nl/en/publications/towards-improved-flood-resilience-integrating-flood-protection-in (Accessed July 28, 2025).

[B94] Wang X. Komatsu S. (2022). The role of phytohormones in plant response to flooding. Int. J. Mol. Sci. 23, 6383. doi: 10.3390/ijms23126383. PMID: 35742828 PMC9223812

[B95] Wang T. Zou Q. Guo Q. Yang F. Wu L. Zhang W. (2019). Widely targeted metabolomics analysis reveals the effect of flooding stress on the synthesis of flavonoids in Chrysanthemum morifolium. Molecules 24, 3695. doi: 10.3390/molecules24203695. PMID: 31615126 PMC6832227

[B96] War A. R. Sharma H. C. Paulraj M. G. War M. Y. Ignacimuthu S. (2011). Herbivore induced plant volatiles: Their role in plant defense for pest management. Plant Signaling Behav. 6, 1973–1978. doi: 10.4161/psb.6.12.18053. PMID: 22105032 PMC3337190

[B97] Weldegergis B. T. Zhu F. Poelman E. H. Dicke M. (2015). Drought stress affects plant metabolites and herbivore preference but not host location by its parasitoids. Oecologia 177, 701–713. doi: 10.1007/s00442-014-3129-x. PMID: 25370387

[B98] Wetzel W. C. Kharouba H. M. Robinson M. Holyoak M. Karban R. (2016). Variability in plant nutrients reduces insect herbivore performance. Nature 539, 425–427. doi: 10.1038/nature20140. PMID: 27749815

[B99] White T. C. R. (1969). An Index to Measure Weather-Induced Stress of Trees Associated With Outbreaks of Psyllids in Australia. Ecology 50, 905–909. doi: 10.2307/1933707

[B100] Wu J.-D. Li J.-C. Wei F.-Z. Wang C.-Y. Zhang Y. Sun G. (2014). Effects of nitrogen spraying on the post-anthesis stage of winter wheat under waterlogging stress. Acta Physiol. Plant 36, 207–216. doi: 10.1007/s11738-013-1401-z. PMID: 41933263

[B101] Xiao Y. Wu X. Sun M. Peng F. (2020). Hydrogen sulfide alleviates waterlogging-induced damage in peach seedlings via enhancing antioxidative system and inhibiting ethylene synthesis. Front. Plant Sci. 11. doi: 10.3389/fpls.2020.00696. PMID: 32547587 PMC7274156

[B102] Xiong Y.-D. Han W.-H. Zhao C. Chi Y.-J. Wang J.-J. Liu S.-S. . (2026). Flooding impairs plant resistance to piercing-sucking insects via ethylene-mediated suppression of callose deposition. Pest. Manage. Sci. 82, 613–624. doi: 10.1002/ps.70221. PMID: 40948149

[B103] Yamauchi T. Noshita K. Tsutsumi N. (2021). Climate-smart crops: Key root anatomical traits that confer flooding tolerance. Breed. Sci. 71, 51–61. doi: 10.1270/jsbbs.20119. PMID: 33762876 PMC7973492

[B104] Yeung E. Bailey-Serres J. Sasidharan R. (2019). After the deluge: Plant revival post-flooding. Trends Plant Sci. 24, 443–454. doi: 10.1016/j.tplants.2019.02.007. PMID: 30857921

[B105] Yeung E. van Veen H. Vashisht D. Sobral Paiva A. L. Hummel M. Rankenberg T. . (2018). A stress recovery signaling network for enhanced flooding tolerance in Arabidopsis thaliana. Proc. Natl. Acad. Sci. 115, E6085–E6094. doi: 10.1073/pnas.1803841115. PMID: 29891679 PMC6042063

[B106] Yuan L.-B. Chen M.-X. Wang L.-N. Sasidharan R. Voesenek L. A. C. J. Xiao S. (2023). Multi-stress resilience in plants recovering from submergence. Plant Biotechnol. J. 21, 466–481. doi: 10.1111/pbi.13944. PMID: 36217562 PMC9946147

[B107] Yuan L.-B. Dai Y.-S. Xie L.-J. Yu L.-J. Zhou Y. Lai Y.-X. . (2017). Jasmonate regulates plant responses to postsubmergence reoxygenation through transcriptional activation of antioxidant synthesis. Plant Physiol. 173, 1864–1880. doi: 10.1104/pp.16.01803. PMID: 28082717 PMC5338657

[B108] Zhao J.-Y. Lu Q. Sun J. Sun L.-Y. Ma R. Wang Y. . (2025). Fall armyworm-induced secondary metabolites in sorghum defend against its attack. Insects 16, 218. doi: 10.3390/insects16020218. PMID: 40003847 PMC11856983

[B109] Zheng Y. Zhang X. Liu X. Qin N. Xu K. Zeng R. . (2021). Nitrogen supply alters rice defense against the striped stem borer Chilo suppressalis. Front. Plant Sci. 12. doi: 10.3389/fpls.2021.691292. PMID: 34381479 PMC8351598

[B110] Zhou W. Chen F. Meng Y. Chandrasekaran U. Luo X. Yang W. . (2020). Plant waterlogging/flooding stress responses: From seed germination to maturation. Plant Physiol. Biochem. 148, 228–236. doi: 10.1016/j.plaphy.2020.01.020. PMID: 31981875

[B111] Zhou S. Richter A. Jander G. (2018). Beyond defense: Multiple functions of benzoxazinoids in maize metabolism. Plant Cell Physiol. 59, 1528–1537. doi: 10.1093/pcp/pcy064. PMID: 29584935

[B112] Zurweller B. A. Motavalli P. P. Nelson K. A. Dudenhoeffer C. J. (2015). Short-term soil nitrous oxide emissions as affected by enhanced efficiency nitrogen fertilizers and temporarily waterlogged conditions. JAS 7, 1. doi: 10.5539/jas.v7n12p1

