# Supplementary material for: Flooding: another abiotic stressor to consider in plant-insect interactions
Source: Front Plant Sci. 2026 Apr 28;17:1813020. doi: 10.3389/fpls.2026.1813020 (PMC13160821; doi:10.3389/fpls.2026.1813020)
Supplement: Supplementary file 1 [file Table1.docx]

**Title - Flooding: Another abiotic stressor to consider in plant-insect interactions.**

**Authors**- Satinderpal Kaur, Esther Ngumbi

Department of Entomology, University of Illinois Urbana-Champaign, Urbana, IL 61801, USA

Corresponding author

Esther Ngumbi

enn@illinois.edu

Supplementary table 1: Studies on flooding-induced changes in crop plants over the past 5 years.

| Sr. No. | Study system | Plant growth stage | Nature of flooding | Parameters studied | Methodology used | Main findings |
| --- | --- | --- | --- | --- | --- | --- |
| 1. (Shang et al., 2025) | Red clover, *Trifolium pratense* L. | When the leaves were 2 cm long | 5 cm water over plants kept in water tank  Soil- nutrient soil: vermiculite: perlite of 3:1:1 | microstructure, physiological indicators, and the key genes and metabolic pathways under submergence stress in the root system | Physiological indices and gene expression | -under submergence, the root system increased the number of root conduits and cortex thickness  -number of down-regulated genes were greater than number of upregulated genes under submergence stress |
| 2. (Wu et al., 2025) | soybean (Glycine max (L.) | R1/R2 | 4-day partial water submergence in field conditions  Soil- silt loamy | To evaluate the grain yield and seed protein and oil content of soybean genotypes under non-flooding and flooding conditions in a 2-year field study | Flood damage score, grain yield | -tolerant, moderate and susceptible genotypes experienced 33%, 44% and 51% yield losses  -no impact of flooding on seed protein and oil content |
| 3. (Novais et al., 2025) | corn (*Zea mays* L.) | V4-V6 | For 4 days using overhead sprinklers | to test how different pre-plant Nitrogen sources and post-emergence waterlogging affect corn N uptake, yield, and efficiency of applied N. | Nutrient concentrations and grain yield | -Waterlogging reduced grain yield by 9% to 25%,  - N source (across waterlogging treatments) increased grain yield over the control plots by 14% to 45% or 236% to 343%, depending on location. |
| 4. (Mignolli et al., 2024) | Tomato *(Solanum lycopersicum L.)* | 10 true-leaf stage | 1 cm water over cotyledonary lobe for 6 days | To understand the effect of hilling in improving plant recovery after flooding | Growth and photosynthetic parameters | Hilled plants were found to recover the root growth, plant nitrogen content and photochemical efficiency |
| 5. (Kaji et al., 2024) | *Arabidopsis thaliana* | 4 week old plants | Submergence was combined with physical water flow | To analyze the growth and transcriptome of plants exposed to submergence or flooding with physical flow. | RNA-seq analysis | -flooding with physical flow reduced the rosette diameters, especially at faster flow rates  -upregulation of SA synthesis, JA synthesis, and ethylene signaling transcripts under flooding with physical flow. |
| 6. (Martins et al., 2024) | soybean (Glycine max (L.) |  | Waterlogging + NaCl in greenhouse conditions | To understand soybean photosynthesis performance during saline waterlogging | Photosynthesis measures | - the combined hypoxia + NaCl treatment resulted in a lower net CO_2_ assimilation rate, ФPSII, and levels of photosynthetic pigments during the waterlogging period  - full recovery was not achieved under any condition during the reoxygenation periods tested |
| 7. (Striesow et al., 2024) | Tomato, *Solanum lycopersicum* |  | trays were filled with distilled water until the quartz sand substrate was covered for 48 hours | To understand the impact of hypoxia applied by waterlogging on root lipid composition | Lipid extraction and transcriptome analysis | -11 lipid species were found exclusively under flooding conditions, mostly belonging to glycerophospholipids and glycerolipids  - an increased level of polyunsaturation was observed in the fatty acid chains |
| 8. (Jahan et al., 2024) | Tomato, *Solanum lycopersicum* | 4- leaf stage | 2-3 cm water level above soil surface for 10 days  Soil- peat:vermiculite 2:1 | To test the efficiency of Melatonin in mitigating the adverse effects of waterlogging | Gas exchange parameters and gene expression | -Melatonin increased the concentrations of ABA and ACC, and decreased the JA, IAA and GA production  -Melatonin increased waterlogging tolerance by up-regulating ethylene biosynthesis gene transcription and inhibiting programmed cell death regulated enzymes. |
| 9. (Luo et al., 2024) | Winter wheat, *Triticum aetivum* | Seedling stage | Waterlogging was stimulated by using artificial rain  Soil- silt loam | Field study to understand plant root traits, soil nitrogen and phosphorous distribution under waterlogging stress | Physical and chemical characteristics of soil | -waterlogging increased root length, surface area and volume  -nitrogen and phosphorous content was higher in upper soil layers and it decreased after 20 cm of soil layer. |
| 10. (Patel et al., 2024) | Tomato, *Solanum lycopersicum* | Flowering stage | submerged potted plants into a tank filled with water for 4 days | To evaluate the efficiency of a commercial formulation, Carrabiitol^®^, an oligosaccharide polyol composition, in alleviating flooding stress | Growth, physiological and biochemical parameters | Carrabiitol^®^ improved the plant growth under flooding conditions by improving gas exchange parameters and antioxidant potential |
| 11. (Wang et al., 2024) | ginger (*Zingiber officinale*) | About 50 cm plant height | Flooding substrate with 2 mm of water film  Soil- coco coir/organic soil mixture, 1:1 | To test the efficiency of Urea peroxide (UHP) in mitigating waterlogging stress | ROS metabolism assay and photosynthetic parameters | -waterlogging reduced the photosynthetic performance and chlorophyll content  -application of UHP improved stress recovery by restoring the physiological activity of root system. |
| 12. (Seymen et al., 2024) | Spinach (*Spinacea oleracea* L.) | 5-week old plants | 2-3 cm of water above soil surface.  Soil- garden clay soil loam containing 2.9 % organic matter | To test the efficiency of melatonin and nitric oxide in mitigating flooding stress | Photosynthetic pigments analysis and antioxidant activity | -The melatonin and nitric oxide improved plant growth in flooding conditions by balancing photosynthetic pigment content  -it also promoted the ROS- scavenging antioxidant defense system. |
| 13. (Cid et al., 2024) | Wheat, *Triticum aetivum* | Third leaf stage | 12 cm above soil surface for 12 days.  Soil- loamy/sandy | To study the metabolic and transcriptomic changes in wheat under waterlogging stress | Transcriptomics and metabolomics | -anaerobic fermentation processes were found as a local response of roots to waterlogging stress  -decreased roots to shoots translocation of nutrients was found  -alanine was found to be most abundant metabolite in xylem exudates. |
| 14. (Shen et al., 2024) | *Hemarthria compressa* | Tillering stage | Plants were placed into a bucket of water.  Soil- vermiculite, vegetative soil, and perlite (3:1:1) | to investigate the gene expression and molecular mechanisms under submergence stress in the root systems of two *H. compressa* genotypes with different submergence stress tolerance levels | transcriptomics | -the genes of several transcriptomic families such as C2H2, bHLH were found to be highly expressed, which might have a adaptive response to submergence stress  - number of adventitious roots and root length was found to be increased after submergence stress. |
| 15. (Teinseree et al., 2024) | Sugarcane, *Saccharum officinarum* | 5 month old | 45 cm of water above soil surface | To study the flooding tolerance in different sugarcane genotypes under recurring floods |  | -different genotypes were found to vary in flooding tolerance  -flooding resulted in increase in cane height but decreased tillering |
| 16. (Seymen et al., 2023) | Onion (*Allium cepa* L.) |  | 2-3 cm of water above soil surface for 10 days | To test the effect of proline and glycine betaine on growth, physiological and biochemical parameters of plants under flooding stress | Growth and physiological parameters | - proline and glycine betaine applications promoted plant growth and reduced negative impacts of flooding |
| 17. (Huđ et al., 2023) | White cabbage, *Brassica oleracea* var. *capitata* |  | 2-3 cm above soil surface | To test the response of single and repeated flooding events on morphological, photosynthetic traits of plants | Photosynthetic and stress parameter analysis | -White cabbage was not found to be very sensitive to short term flooding, but repeated flooding showed signs of stress  -no any effect on morphological or photosynthetic parameters was found, suggesting this plant might be tolerant to flooding. |
| 18. (Gong et al., 2022) | Hot pepper (*Capsicum annuum* L.) | 150 days age | 2 cm above the top of the plastic pot | To understand the molecular mechanisms in waterlogging tolerance in hot pepper | Gene expression | -Stomatal conductance was found to be reduced after flooding, but no effect on photosynthetic activity was found  - antioxidase genes, cell wall synthesis pathway genes, and calcium ion regulation pathway genes were found to be altered by flooding stress. |
| 19. (Mamun et al., 2022) | Soybean, *Glycine max* | Flowering stage | 5 cm of water above the ground level | Field study to understand the effect of potassium on plant growth and yield under waterlogging conditions |  | -waterlogging decreased the seed oil content and absorption of nitrogen, phosphorus, and potassium  -a split application of potassium after the flooding water recedes might be efficient in improving grain yield in flooding affected plots. |
| 20. (Francioli et al., 2022) | Wheat, *Triticum aestivum* L. | tillering, booting, or flowering stage | 5 cm of water above the soil surface for 12 days  Soil- sandy loam | To study the response of wheat microbiota to flooding stress | DNA extraction and characterization | -flooding reduced plant fitness and caused shift in the microbiota assembly  fungal pathogens associated with important cereal diseases, such as *Gibberella intricans*, *Mycosphaerella graminicola*, *Typhula incarnata* and *Olpidium brassicae* were found to increase in abundance under flooding |
| 21. (Stasnik et al., 2022) | Camelina, *Camelina sativa* | 7-9 leaves and 15-16 leaves plants | Water 1 cm above the soil level in pots Soil- soil peat substrate | To study the physiological responses of different lines of Camelina to waterlogging stress |  | -younger plants were found to be more susceptible to flooding than older plants  -waterlogging reduced yield of the plants, but yield reduction was different for different lines of Camelina. |
| 22. (Park et al., 2022) | Sweet Potato, *Ipomoea batatas* (L.) Lam | 2 weeks old plants | water was added to the pots to submerge approximately 65% of the aboveground tissue | To characterize the regulation of ethylene (ET), reactive oxygen species (ROS), and nitric oxide (NO) metabolism under early flooding stress | Gene expression | -as a result of flooding, levels of respiratory burst oxidase homologs and metallothionein-mediated ROS scavenging were found to be increased  -the genes involved in NO biosynthesis and scavenging were also found to be increased. |
| 23. (Daku et al., 2022) | maize (*Zea mays L.*) | V6, VT and R3 stage | 0 cm, 2–3 cm, and 7–8 cm of water above soil surface | Field experiment to test the effect of temporary flooding on growth and productivity |  | -at tasseling stage, the 3-day and 6-day flooding was found to reduced yield by 35%  -at V6 stage, 4-6 day flooding reduced yield by 21%. |
| 24. (Wang et al., 2022) | Soybean, *Glycine max* | R1 | 2–3 cm water above the soil surface  Soil- cultivated soil, humus soil, and sand in a ratio of 7:2:1 | To study the effect of flooding on leaf physiology and yield of soybean and to test the effect of uniconazole in mitigating plant stress response |  | -waterlogging increased antioxidant enzyme activity  -uniconazole improved the antioxidant defense mechanisms and hence promoted yield of plants under waterlogging stress. |
| 25. (Cotrozzi et al., 2021) | Durum wheat, *Triticum turgidum* L. subsp. *durum* | Tillering stage | 1 cm of water was maintained above the soil surface  Soil- sandy loam | To evaluate the impact of two durations of flooding on physiological, biochemical, biometric, and yield parameters |  | -at tillering stage, the flooding reduced photosynthetic activity which in turn reduced tiller formation and impacted crop yield |
| 26. (Tamang et al., 2021) | Soybean, *Glycine max* | V1 stage | Flooding for 3-6 days | To study overlapping and stress specific hormonal responses to flooding, drought and its recovery | Transcriptomics and metabolomics | - positive regulation of trehalose and sucrose metabolism and negative regulation of cellulose, tubulin, photosystem II and chlorophyll synthesis was found in both stresses  -downregulation of distinct pathways was found for saving energy in case of each stress  - abscisic acid and ethylene responses were activated in common under both stresses |
| 27. (Li et al., 2021) | Rapeseed, *Brassica napus* L. | Seeds with radicle 2-5 mm long | Seeds placed in double-distilled water in sealed tube (10 mL) | To characterize the transcriptomic differences in two lines of rapeseed under flooding at germination stage | Gene expression | - the processes such as “hormone-mediated signaling pathway”, “response to organic substance response”, “motor activity”, and “microtubule-based process” are likely to confer rapeseed flooding resistance |
| 28. (Zhou et al., 2021) | Soybean, *Glycine max* | R1 | 5-10 cm of flooding water for 8 days in field | Field study- testing the efficiency of UAV- based image system to estimate flood induced plant injuries |  | The proposed method was promising in estimating flooding injury score in soybean |
| 29. (Choi et al., 2021) | common buckwheat, *Fagopyrum esculentum* cv. Harunoibuki) | 17, 30, and 42 days after sowing | 5 cm of water depth for 3 days | To test what duration of waterlogging and at what stage has severe effects on yield |  | -waterlogging influenced plant height, SPAD (soil plant analysis development) value, chlorophyll fluorescence, root analysis (length, surface area, and volume), and dry weight of plants  -waterlogging impacted root parameters more than shoot parameters  -early stage plants were found to be more susceptible to flooding damage |
| 30. (Chen et al., 2020) | Durum wheat, *Triticum durum* Desf. | 20-day old seedlings | By adding water (2-L in each pot) above the soil surface to immerse seedlings for seven days  Soil- ature manure:organic soil:peat, 1:1:1 | To investigate the changes in phenolic compounds in leaves under flooding and ozone stress |  | - Different phenolic compounds were found in flooded plants and ozone stressed plants, suggesting that phenolic response to environmental constraints is stress specific |
| 31. (Zhu et al., 2020) | Rice, *Oryza sativa* L. | Jointing-heading stage | Different combinations of drought and flooding | To study the cumulative effect of drought and flooding on photosynthetic ability of rice | Stomatal conductance, photosynthesis and transpiration measurements | The results showed photosynthetic inhibition under stress combination treatment |
| 32. (Hashimoto et al., 2020) | Soybean | seedling | Flooding water upto 4 cm in seedling tray | Test the effect of silver nanoparticles mixed with inorganic chemicals to relive flooding stress | proteomics | silver NPs, nicotinic acid, and KNO_3_ improved soybean growth under flooding stress by regulating protein quality |
| 33. (Tian et al., 2020) | Spring maize | V3, V6 and VT | 3, 6 and 9 days of flooding,  5, 10 and 15 days of waterlogging in field | To test the adverse effects of flooding on stem strength and crop yield | Fluorescence microscopy for stem microstructure | Flooding reduced lodging ability of stem and reduced crop yield |
| 34. (Lothier et al., 2020) | *Medicago truncatula* | 3 weeks after germination | 7 and 21 days of flooding by filling pots with nutrient solution | To monitor changes in the metabolome of roots and shoots under flooding stress | Metabolomics | -shoots showed higher concentration of sugar and starch, while their concentration decreased in roots, suggesting inhibition of sugar movement. |
| 35. (Rastogi et al., 2019) | *Ocimum tenuiflorum* | 2 months old plants | 0.5 inches of water above soil level for 30 days | To test the pathway gene expression changes under abiotic stress | Metabolmics, transcriptomics | Identified several stress tolerance genes |
| 36. (Ruperti et al., 2019) | Grapes, *Vitis vinifera* | 3 years old plants | Water maintained 5 cm above soil surface for 2, 8, 16 and 21 days | To characterize the metabolic and transcriptional influence of flooding on grapes | Untargeted transcriptomic and metabolic profiling | Flooding resulted in an increase in hypoxia inducible metabolites and changes in metabolic pathways |
| 37. Gu et al., 2019 | Oilseed rape, *Brassica napus* | 5 true- leaves stage | 1 cm of water over soil surface for 3, 6 and 9 days | To study the influence of flooding on soil nutrients and enzyme activity | Mineral nutrient concentration and soil enzyme activity | Flooding influenced the N and P concentrations in plants tissues as well as soil, suppressed the activities of soil enzymes |
| 38. (Huang et al., 2019) | Rice, *Oryza sativa* L. | Jointing-booting stage | 5-6 days of complete submergence | To study root growth dynamics and yield of rice under drought-flood alternating stress | N/A | The drought and flooding have interactive effects on root growth, but flooding showed highest reduction in yield than individual stresses |
| 39. (Barickman et al., 2019) | *cucumber (Cucumis sativus L.)* | 2 weeks after emergence | 10 cm water above pot surface for 10 days | to measure the photosynthetic and key metabolites of cucumber plants under waterlogging conditions | Colorimetric analysis of all photosynthetic pigments | Waterlogging reduced photosynthesis parameters and plant growth traits |
| 40. (Anee et al., 2019) | *Sesamum indicum* | 21 days after sowing | 2 cm of standing water over surface for 2, 4, 6 and 8 days | To study the effect of different duration of flooding on photosynthetic and biochemical pathways |  | Flooding reduced the photosynthetic pigments, but increased the enzymatic activity for oxidative scavenging |
